# The transition of youth with diabetes from paediatric to adult diabetes care services: a consensus report of the joint working group of the International Society for Pediatric and Adolescent Diabetes (ISPAD), the European Association for the Study of Diabetes (EASD) and the American Diabetes Association (ADA)

**DOI:** 10.1007/s00125-026-06803-5

**Published:** 2026-09-14

**Authors:** Carine de Beaufort, Steven James, Jill Weissberg-Benchell, Apoorva Gomber, Laura Cudizio, Nisa M. Maruthur, Sarah K. Lyons, Frank Snoek, Sze May Ng, Aleksandra Araszkiewicz, Eva Hagström Toft

**Affiliations:** 1 Faculty of Science, Technology and Medicine, University of Luxembourg, Esch-Belval, Luxembourg; 2 OBEDIA-Kids, Centre Hospitalier de Luxembourg, Luxembourg, Luxembourg; 3 Department of Pediatrics, University Hospital of Brussels, Brussels, Belgium; 4 School of Health, University of the Sunshine Coast, Petrie, QLD Australia; 5 Department of Medicine, University of Melbourne, Parkville, VIC Australia; 6 School of Medicine, Western Sydney University, Campbelltown, NSW Australia; 7 Pritzker Department of Psychiatry and Behavioral Health, Ann and Robert H. Lurie Children’s Hospital, Chicago, IL USA; 8 Division of Global Health Equity in the Department of Medicine, Brigham and Women’s Hospital, Boston, MA USA; 9 Santa Casa of São Paulo, São Paulo, Brazil; 10 Division of General Internal Medicine, Johns Hopkins University School of Medicine, Baltimore, MD USA; 11 Department of Pediatrics, Baylor College of Medicine, Houston, TX USA; 12 Department of Medical Psychology, Amsterdam UMC, Vrije Universiteit Amsterdam, Amsterdam, the Netherlands; 13 Faculty of Health, Social Care and Medicine, Edge Hill University, Ormskirk, UK; 14 Department of Paediatrics, Mersey and West Lancashire Teaching Hospitals NHS Trust, Ormskirk, UK; 15 Department of Internal Medicine and Diabetology, Poznan University of Medical Sciences, Poznan, Poland; 16 Department of Clinical Science and Education, Karolinska Institute at Södersjukhuset, Stockholm, Sweden; 17 Ersta Hospital, Stockholm, Sweden

**Keywords:** Adolescent, Adult, Diabetes, Paediatric, Transition of care youth

## Abstract

Young adulthood is a period of significant and unique physiological, psychological, sexual and social upheaval. Related challenges can have an impact on effective diabetes self-management, with high rates of unplanned healthcare use recorded for many, and consequent increases in the risks of premature morbidity and mortality. Healthcare professionals need guidance in delivering transition support for adolescents and young adults with diabetes. This would help to facilitate the formulation and implementation of local policies and procedures, enabling guidelines to be translated into practice and improving transition of care readiness. This led ISPAD, EASD and ADA to convene a working group to produce a consensus report on the transition from paediatric to adult diabetes healthcare services. The ACCORD and AGREE II recommendations for the development of consensus reports and clinical guidelines, respectively, were followed. Existing diabetes-specific recommendations on the transition of care, published statements, a prior position paper, general recommendations and online toolkits were initially identified and reviewed. Current evidence was then systematically reviewed, supplemented with primary data on the perceptions and experiences of healthcare professionals and of people with diabetes and their caregivers from global surveys. Evidence with a grading of A or B was automatically included in the consensus statements; evidence with a C or E grading was subjected to a Delphi survey for validation. Consensus was obtained for 31 statements on the full transition of care continuum and the three unique stages of pre-transfer, transfer and post-transfer. This consensus report should be viewed as a guide and adjusted to local settings.

**Graphical Abstract:**

**Figure:**
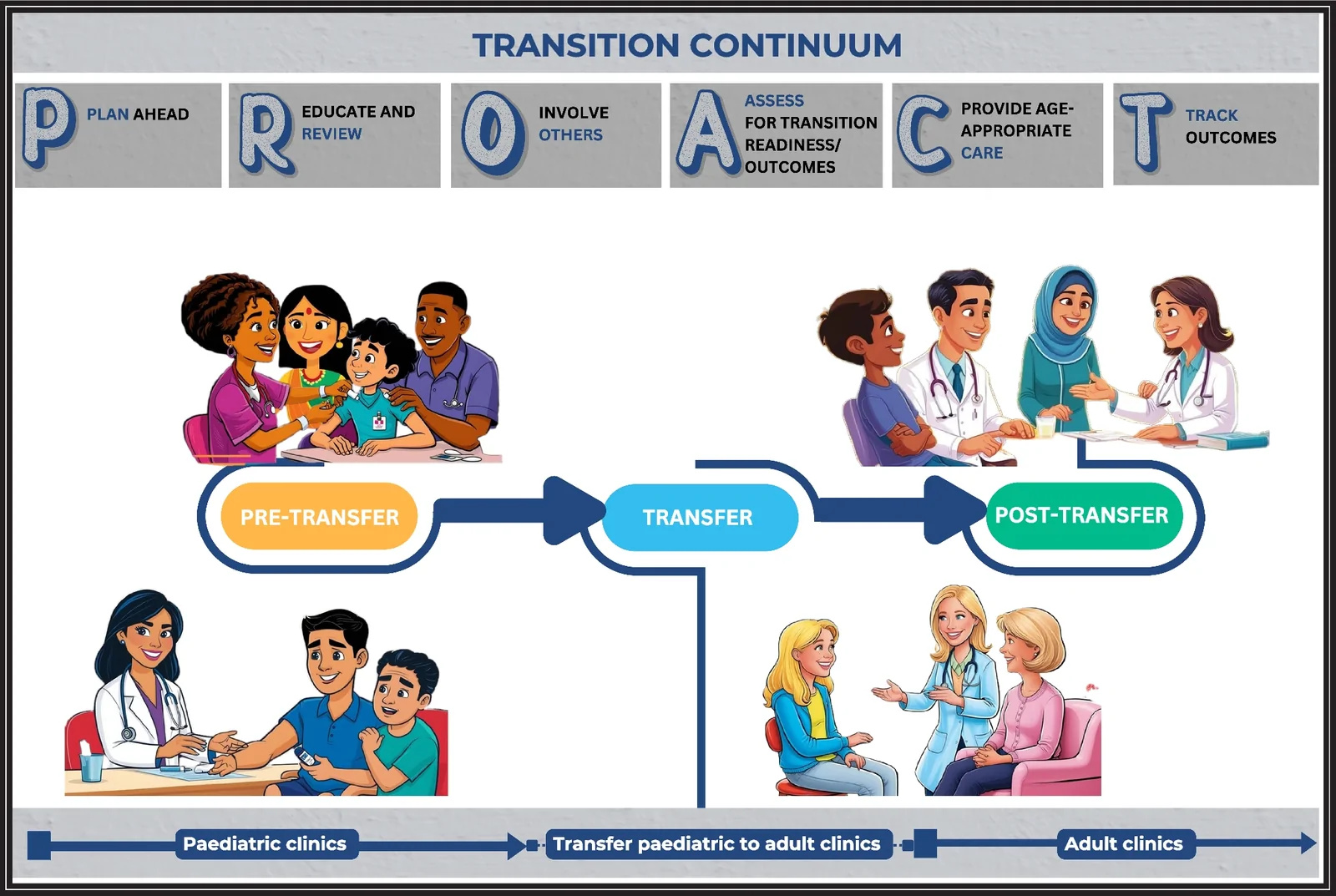

**Supplementary Information:**

The online version contains peer-reviewed but unedited supplementary material available at https://doi.org/10.1007/s00125-026-06803-5.

## Background and rationale

Young adulthood can be a period of significant and unique physiological, psychological, sexual and social upheaval [1–3]. The challenges of becoming an adult, coupled with an actively developing prefrontal cortex, which is crucial for complex functions such as decision-making, planning and impulse control, can impact on effective diabetes self-management and consequently diabetes-related outcomes. Healthcare services for young adults with diabetes, which are key to support diabetes self-management and early complication detection and treatment, are inconsistent [4–6]. This is observed worldwide. High rates of missed healthcare contacts, emergency service use and inpatient admissions have been observed in the period of transition from paediatric to adult care [4, 5, 7], with consequent increases in risks of premature morbidity and mortality [4, 5, 8–14].

Adolescents and young adults with diabetes need uninterrupted access to specialised medical care, peer support, and safe school and work environments to enable them to gain the confidence and skills needed to independently manage their disease. Negative health outcomes in young adulthood may, in part, occur due to unsuccessful transition from paediatric to adult-based diabetes healthcare services [15, 16]. Transition of care is defined as the purposeful, planned process of adolescents and young adults with chronic physical and medical conditions from child-centred to adult-oriented healthcare systems [17]. This transition typically starts in early adolescence and ends during the stage of emerging adulthood. It is more than the simple transfer of a medical file or a transfer letter; instead, transition of care should be planned, prepared and individualised, based on constant and constructive collaboration between the person with diabetes, their caregivers, and the paediatric and adult diabetes healthcare teams [3, 17]. Before the actual transfer of a person with diabetes (pre-transfer) from (specialised) paediatric care to adult care, attainment of the knowledge and skills important for this transfer and integration into the adult healthcare system is vital. This transition readiness helps determine whether transition completion (post-transfer) will be successful, with positive integration into adult care, both specialised and general medical practice.

Healthcare professionals and services need guidance in delivering support around the transition of care. This would help to facilitate the formulation and implementation of local policies and procedures, enabling guidelines to be translated into practice and promoting transition readiness. Given the heterogeneity of diabetes care models worldwide and the multitude of factors that may influence the transition process, including age, financial resources, preferences of people with diabetes and their caregivers, healthcare delivery and access, and national policies, differences in transition models are to be expected [18–22]. Online educational resources for transition of care may exist in some countries and regions but are absent in others [23]. Collectively, this led ISPAD, EASD and ADA to convene a working group of specialists in this field to develop a consensus report on transition from paediatric to adult diabetes healthcare services, ensuring that the perceptions of paediatric- and adult-based healthcare professionals and people with diabetes and their caregivers worldwide were incorporated. The main objective of this consensus report was to inform healthcare professionals on the best possible and most acceptable way to diagnose and treat certain diseases, and address a decision-making area [23].

## Methodology

To develop the consensus report, the working group met monthly via videoconference and had a face-to-face meeting in Florence, Italy (2024); the ACCORD recommendations for the development of consensus reports and AGREE II recommendations for the development of clinical guidelines (see electronic supplementary material [ESM] 1 and 2) were followed [23, 24]. Further details on the roles and areas of expertise or experience of the working group are provided in ESM 1.

**Fig. 1:**
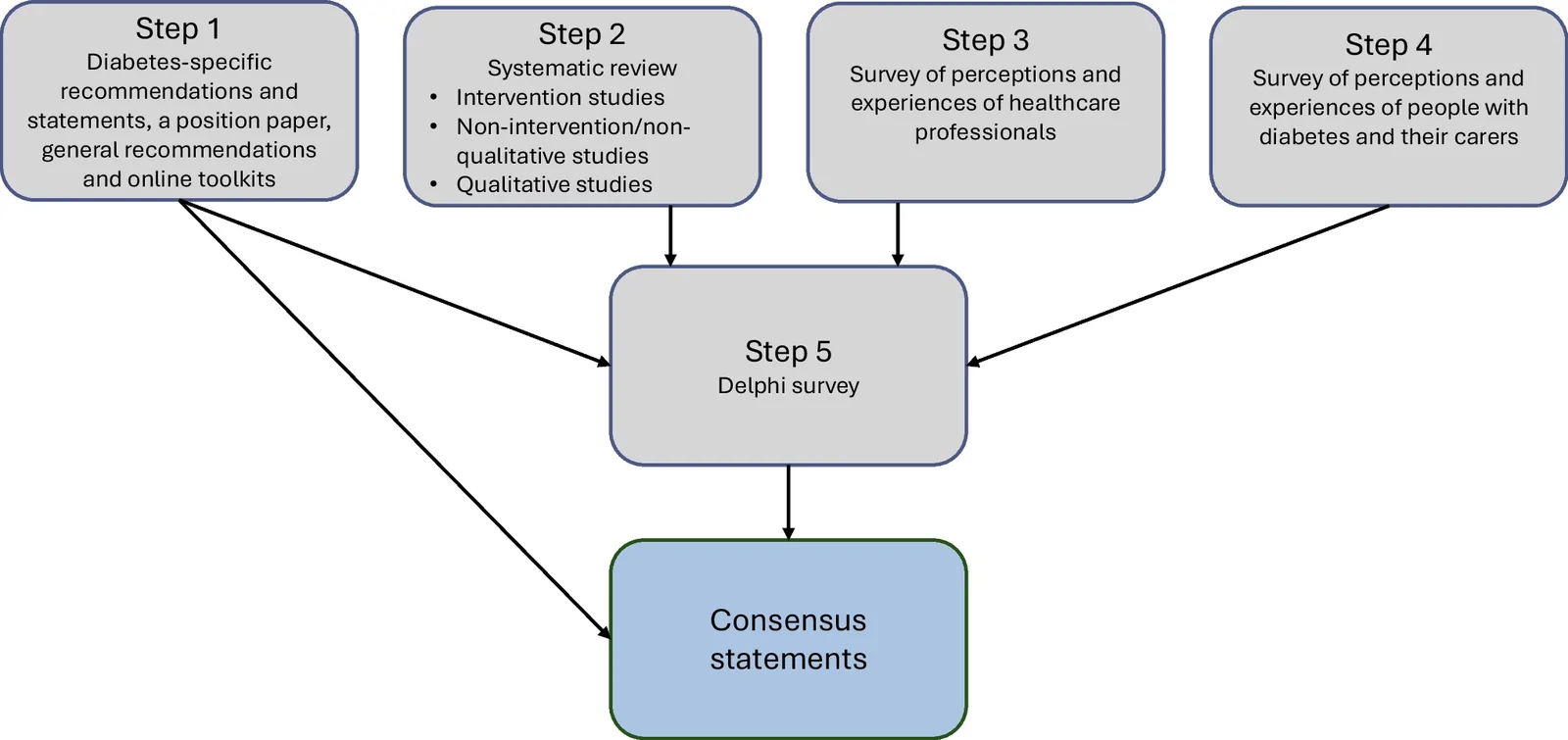
Procedures used to develop the consensus statements for transition from paediatric to adult diabetes healthcare services

As a starting point, existing diabetes-specific recommendations on the transition of care reported in guidelines [3, 25–27], a prior position statement [8], general recommendations [17, 28, 29] and online toolkits [3, 30–33] were identified and reviewed (Fig. 1). To help determine further consensus statements, a systematic review of the current evidence relating to transition readiness and transfer of care outcomes was conducted. This yielded two publications, one focusing on intervention studies [34] and the other on non-intervention/non-qualitative studies [35], with a further publication focusing on qualitative studies planned. We supplemented these data with data on the perceptions and experiences of healthcare professionals [36] and of people with diabetes and their caregivers [37] obtained through global surveys (Fig. 1). Grading of evidence followed recommendations reported by the ADA [38]. The ADA’s grading system uses A, B, C or E to illustrate the evidence level that supports each recommendation. A-level evidence is clear evidence from well-conducted, generalisable RCTs that are adequately powered; B-level evidence is supportive evidence from well-conducted cohort studies; and C-level evidence is supportive evidence from poorly controlled or uncontrolled studies. E-level evidence is expert consensus or clinical experience and is a separate category for recommendations for which there is no evidence from clinical trials, clinical trials may be impractical or there is conflicting evidence. Recommendations supported by a level of evidence of A or B were automatically included in the consensus statements, whereas recommendations with a level of evidence of C or E (which did not meet A or B evidence grading) were subjected to a Delphi survey for validation [23, 39, 40]. Further details on these elements are provided below.

### Systematic review

Defined databases were searched for peer-reviewed articles related to transition readiness or actual transfer from paediatric to adult diabetes healthcare services published between January 2018 and December 2023. Studies were included if they were peer-reviewed, were in English, were intervention, non-intervention or qualitative studies related to either transition readiness or transfer to adult diabetes healthcare services, and focused on type 1 or type 2 diabetes. Studies that focused on general chronic illness were eligible for inclusion only if people with diabetes were explicitly included. The studies were reviewed and categorised as intervention studies (*n*=26) [34], non-intervention/non-qualitative studies (*n*=33) [35] or qualitative studies (*n*=24) (publication planned). The protocols for the two systematic reviews have not been published or pre-registered. Despite their publication in peer-reviewed journals [34, 35], and the reported use of rigour in the review methods, omission of pre-registration of systematic reviews can create risks of bias, including selective inclusion and post hoc decision-making. AMSTAR2 checklists, a critical appraisal tool for systematic reviews, including those involving randomised or non-randomised studies of healthcare interventions, are provided to give additional details on the two studies (see ESM 3 [34] and ESM 4 [35]) [41]. For the quality assessment of all studies meeting the inclusion criteria, the Joanna Briggs Institute Checklist for Systematic Reviews was used as a critical appraisal tool [42]. The outcome of the systematic review is discussed below. As none of the studies was excluded irrespective of quality metrics, this may impact the credibility and strength of the consensus statements when based on these studies.

Among the 26 intervention studies, only one reported on an RCT [34]. Most studies were pre–post studies evaluating the impact of transition of care programmes, with most interventions having no benefit on metabolic outcomes and showing variable improvement in care satisfaction and transition readiness. Quality metrics were lacking in some studies, as well as important contextual factors including race and ethnicity. Long-term follow-up was not provided for any of these studies.

Similarly, among the 33 non-intervention/non-qualitative studies, 11 directly measured transition readiness and found associations with constructs including age, glycaemic outcomes and family and peer support [35]. Further, it was also found that older age at transfer was associated with less acute service use, whereas the presence of a mental health disorder while receiving paediatric care was associated with less consistent follow-up post-transfer. In addition, more paediatric visits prior to transfer was associated with a shorter gap between paediatric and adult care and more frequent follow-up post-transfer, whereas parental involvement, promotion of health self-efficacy and meeting the adult team before transfer were associated with improved post-transfer outcomes. Finally, healthcare professional perspectives on impediments to transfer included a long therapeutic relationship and lack of a transition of care protocol.

### Perceptions and experiences of healthcare professionals

Data were collected via an online global survey (seven language options), broadly advertised by ISPAD and EASD, research team members and partners via newsletters, websites, emails and social media [36]. Respondents (*n*=372) were mainly physicians (74.5%), practising in government-funded (59.4%), paediatric (54.0%) and metropolitan (85.8%) settings in Europe (44.9%); 37.1% of respondents were from low- and middle-income countries. Findings demonstrated that few centres used a transition readiness tool (32.8%), had dedicated transition personnel (23.0%) or provided the receiving team with written information (29.6%). Access to psychological support (25.8%), combined (35.2%) or dedicated (34.9%) transition clinics, and structured transition education programmes (22.5%) was limited. Most (91.9%) respondents reported barriers that prevented them from providing a good transition experience. Compared with healthcare professionals from high-income countries, healthcare professionals from low- and middle-income countries expressed a need for more funding (*p*=0.01), a structured protocol (*p*<0.001) and education (*p*<0.001) to address the challenges identified.

### Perceptions and experiences of people with diabetes and their caregivers

Data were collected via an online global survey (seven language options), broadly advertised by ISPAD and EASD, research team members and partners via newsletters, websites, emails, and social media [37]. The target population was limited to people with diabetes aged between 14 and 25 years (or their caregivers). Overall, 90 (61.6%) people with diabetes (mean ± SD age 18.5±3.6 years; diagnosed at 9.0±4.4 years) and 56 (38.4%) caregivers responded to the survey, based in 29 different countries. Higher care satisfaction was reported by those still attending paediatric clinics (58.2%) than those attending adult clinics, as well as more frequent appointments (*p*<0.05); a fixed transfer age (≥18 years) was reported by 65.1% of respondents. Overall, 45.2% reported transfer-related concerns and <50% felt that their psychosocial needs were adequately met. Almost 25% felt unprepared for some areas of self-management. Combined paediatric and adult diabetes clinics (56.2%) and psychological support (50.7%) were the care services most frequently requested.

The Human Research Ethics Committee of the University of the Sunshine Coast, Australia, reviewed and approved the studies on the perceptions and experiences of healthcare professionals and of people with diabetes and their caregivers (approval no.: A231935). Participants provided their informed consent electronically.

### Delphi survey

After reaching consensus on the statements within the working group, based on existing transition guidance [8], the systematic review [34, 35] and the surveys of healthcare professionals [36] and people with diabetes and their caregivers [37], a Delphi panel was established. Panellists were identified through the three diabetes societies (ISPAD, EASD and ADA) based on their contributions to the field of diabetes-related transition (publications), professional diversity and geographical setting. The panel also included members of the #dedoc online diabetes community (https://www.dedoc.org/), ensuring that the perspectives of people with diabetes and their caregivers were also captured. Invitations including an explanation of the topic and a request for demographic information were sent by email to 82 people (physicians: paediatric 35.4%, adult 25.6% and general practitioner 2.4%; nurses: 8.5%; psychologists: 8.5%; dietitians: 1.2%; people with diabetes: 17.1%; WHO representative: 1.2%), of whom 57 (69.5%) accepted (Table 1). A link to the survey including 26 transition-related statements was emailed to panellists on three occasions; 44 (77%) completed the survey, with >90% endorsement obtained for all items [23, 39, 40]. Where panellists provided comments, these were reviewed and discussed by the working group and included where appropriate based on consensus.

**Table 1:** Delphi panel composition

Healthcare profession	*n* (%)
Physician	
Paediatric	23 (40.4)
Adult	9 (15.8)
General practitioner	1 (1.8)
Allied healthcare professional	
Nurse	6 (10.5)
Psychologist	5 (8.8)
Dietitian	1 (1.8)
Person with diabetes/other	
Person with diabetes, family member or advocate	12 (21.1)
Total	57 (100)

## Results

The formulated consensus statements for transition from paediatric to adult healthcare services, their sources and the applicable evidence grading are summarised in Table 2. While statements 1–11 recognise that there are facets of diabetes healthcare that belong to the transition of care continuum, the remaining statements have been subdivided into three different phases: pre-transfer, transfer and post-transfer. Based on the comments of the Delphi panellists, the term ‘healthcare provider’ was changed to ‘healthcare professional’.

**Table 2:** Consensus statements for transition from paediatric to adult diabetes healthcare services, and grading of evidence

No.	Source	Consensus statement	Evidence level
Full transition continuum			
1	Existing evidence base	Diabetes care should be individualised and developmentally appropriate, emphasising adherence to diabetes self-management and consistent use of glucose-lowering medications, to prevent both acute and long-term complications of diabetes [8, 26, 43]	B
2	Existing evidence base	Healthcare professionals should recognise the vulnerability of emerging young adults with diabetes, who may struggle with consistent healthcare access and adherence to diabetes management due to various psychosocial, educational and vocational challenges. These difficulties can lead to worsening glycaemic outcomes. During transfer, both paediatric and adult care professionals should offer support and connect people with diabetes with resources that could benefit them [8]	B
3	Existing evidence base	At diagnosis and during routine follow-up care, youth with diabetes should be screened for psychosocial concerns (e.g. diabetes distress, depressive symptoms and disordered eating behaviours), family factors and behavioural health concerns that could impact on diabetes self-management, using age-appropriate standardised and validated tools. Refer to a qualified behavioural health professional, preferably experienced in childhood diabetes, when indicated [44]	B
4	Existing evidence base	Screening for micro- and macrovascular risk factors should be performed and treatment implemented according to ISPAD, EASD and ADA recommendations [8, 45–48]	A/B
5	Existing evidence base	Age-appropriate monitoring of the mental health of people with diabetes should be an ongoing part of diabetes care (at least annually), even in those who currently have good psychological resources [44, 46]	B
6	Endorsed by Delphi panellists	Healthcare professionals should empower people with diabetes through education and resources that promote diabetes self-management, which should include understanding of their individual needs [26, 36, 37, 49]	E
7	Endorsed by Delphi panellists	Healthcare professionals should assess and help address psychosocial challenges that may impact on diabetes healthcare engagement during the transition period. This may include support for managing stress, peer relationships and lifestyle changes [36, 37, 44, 50, 51]	E
8	Endorsed by Delphi panellists	Healthcare professionals should encourage people living with diabetes to continue to take an active role in their care as they transition to adulthood [36, 37]	E
9	Endorsed by Delphi panellists	Healthcare professionals should encourage continued family involvement, while stimulating the conversation on progressively handing over responsibility of diabetes self-management [15, 36, 37, 52, 53]	E
10	Endorsed by Delphi panellists	Healthcare professionals should empower people living with diabetes to navigate the health system/resources that can help them reconnect with care if they are lost to follow-up. This should include access to medical emergency systems [36, 37]	E
11	Endorsed by Delphi panellists	Birth control, pregnancy planning and risks, use of alcohol and drugs, smoking and driving should be discussed and assessed, with an emphasis on the interplay with diabetes [36, 37]	E
Pre-transfer			
12	Endorsed by Delphi panellists	Paediatric healthcare professionals should develop a transition of care policy/guide specific to their centre/clinic, which should include its approach to transition preparation and transfer to adult care. This process should be shared early, and the contents must be agreed on with people with diabetes and their caregivers [3, 17, 26–28, 36–38, 54]	E
13	Endorsed by Delphi panellists	Paediatric healthcare professionals should identify the roles and responsibilities related to transition preparation of each member of the multidisciplinary diabetes professional team (e.g. diabetologist, diabetes educator, dietitian, social worker and psychologist) [36]	E
14	Endorsed by Delphi panellists	Paediatric healthcare professionals should integrate transition-related topics into ongoing diabetes education in an age-appropriate and developmentally appropriate way [3, 17, 36]	E
15	Endorsed by Delphi panellists	The conversation around transition should be timely (in most cases it should begin at least 12–24 months before transfer) [17, 27, 36, 37, 49]	E
16	Endorsed by Delphi panellists	Paediatric healthcare professionals should assess transition-related knowledge and skills and provide targeted and personalised education on identified gaps [36]	E
17	Endorsed by Delphi panellists	Use of a transition readiness checklist is a helpful way to ensure that comprehensive education and assessment is completed prior to transfer [36, 37]	E
18	Endorsed by Delphi panellists	Paediatric healthcare professionals should invite people with diabetes to ask transfer-related questions and discuss any concerns [27, 36, 37]	E
Transfer			
19	Endorsed by Delphi panellists	During the transfer of care, it is advisable to schedule the first appointment with the adult healthcare professional within 3–4 months of the final paediatric or joint paediatric–adult clinic visit, or assist the person with diabetes to do so [26, 27, 36–38, 55]	E
20	Endorsed by Delphi panellists	A directory of adult healthcare professionals with expertise in caring for young adults may be beneficial for the person with diabetes [36]	E
21	Endorsed by Delphi panellists	Joint clinics with paediatric and adult healthcare professionals should be provided if possible to ensure optimal communication and seamless transition [36, 56]	E
22	Endorsed by Delphi panellists	The use of a dedicated professional (e.g. care navigator, transition coordinator, care ambassador or transition nurse) to assist with the transfer of care (scheduling, follow-up, and linking people with diabetes to adult care services) can facilitate the transfer and prevent gaps in care [36, 57]	E
23	Endorsed by Delphi panellists	Communication between paediatric and adult healthcare professionals should be timely for a smooth transfer (e.g. including key information on medication and treatment plans, laboratory test results and complications) [26, 36–38]	E
24	Endorsed by Delphi panellists	A written medical summary including diabetes history, any complications and current management should be provided to the adult healthcare professional prior to transfer, in agreement with the person with diabetes [27, 36]	E
25	Endorsed by Delphi panellists	Paediatric and/or adult healthcare teams should be prepared to discuss issues around, and inform people with diabetes about, the transfer to adult care (e.g. possible financial costs, availability of medicines, and insurance) [36, 37]	E
26	Endorsed by Delphi panellists	Paediatric healthcare professionals should encourage people with diabetes to connect with peers and engage in peer support activities [36, 37, 58–62]	E
Post-transfer			
27	Endorsed by Delphi panellists	Knowledge gaps and needs with respect to diabetes self-management skills should be assessed and addressed [36, 37]	E
28	Endorsed by Delphi panellists	Psychosocial health should be assessed and addressed [36, 37, 44, 51]	E
29	Endorsed by Delphi panellists	Practical knowledge around access to both specific and general emergency care should be assessed [36, 37]	E
30	Endorsed by Delphi panellists	Visits should be scheduled at least every 3 months during the first year after transfer to adult care, to minimise the risk of loss to follow-up [36]	E
31	Endorsed by Delphi panellists	Telehealth and virtual care can be offered in addition to traditional in-person visits to improve communication with healthcare professionals and provide flexibility of access to care for young adults with high levels of digital literacy [36, 63, 64]	E

**Fig. 2:**
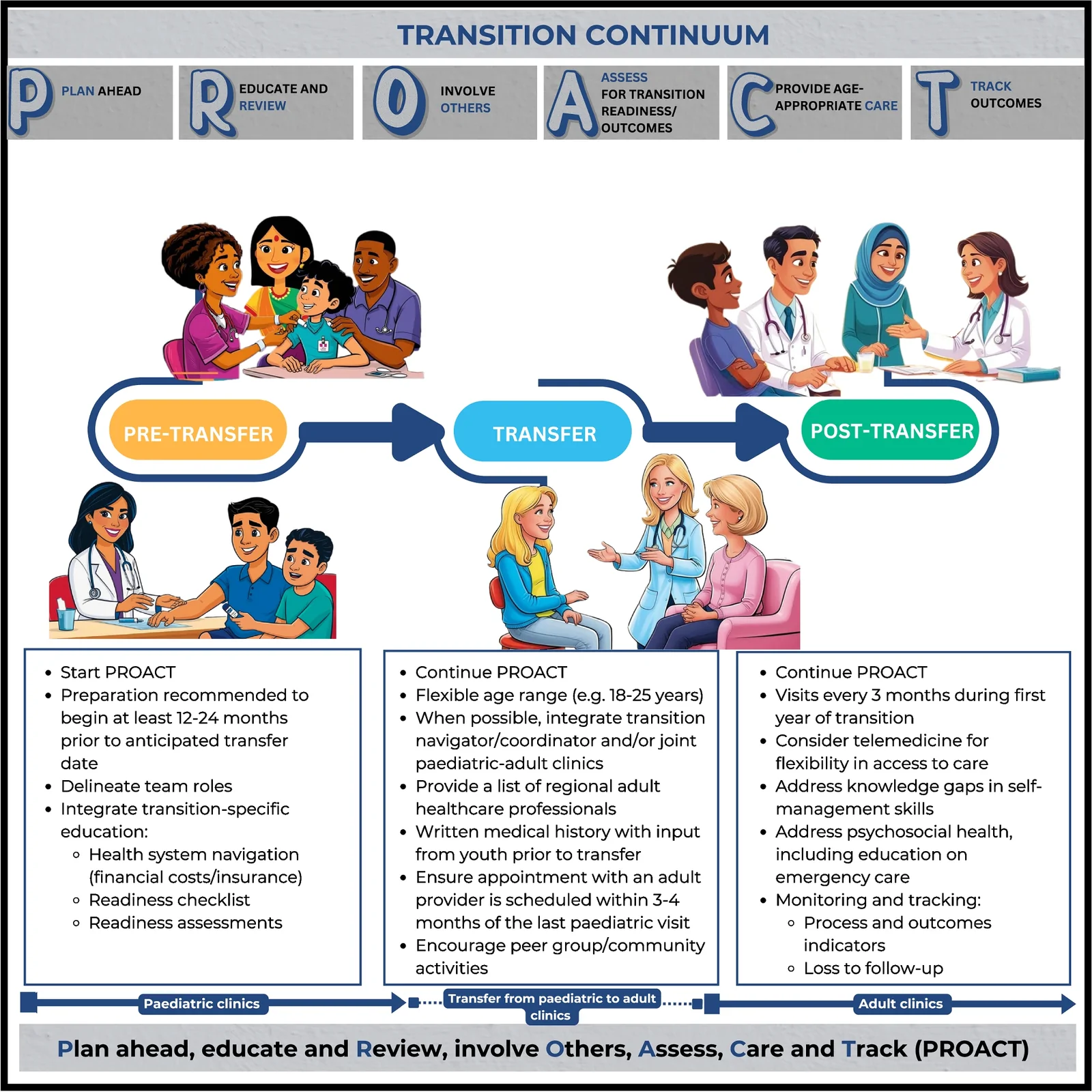
The transition of care continuum. Proposed strategies for transition from paediatric to adult-based diabetes care, detailing the essential steps and distinctions pertaining to pre-transfer, transfer and post-transfer and their intended goals (see readiness checklist and assessment tools [31, 33, 65–67]). The illustrative elements were created using Canva Pro AI (www.canva.com/pro/) to reflect diversity across people and healthcare settings

The proposed strategies for transition from paediatric to adult diabetes care are summarised in Fig. 2. These strategies should, at all stages, include timely Planning (P: Plan ahead), ongoing education and Review (R: educate and Review), the inclusion of Other partners (O: involve Others), the Assessment of transition readiness (A: Assess for transition readiness/outcomes), age-appropriate diabetes Care (C: provide age-appropriate Care) and ongoing outcome evaluation (T: Track outcomes).

**Fig. 3:**
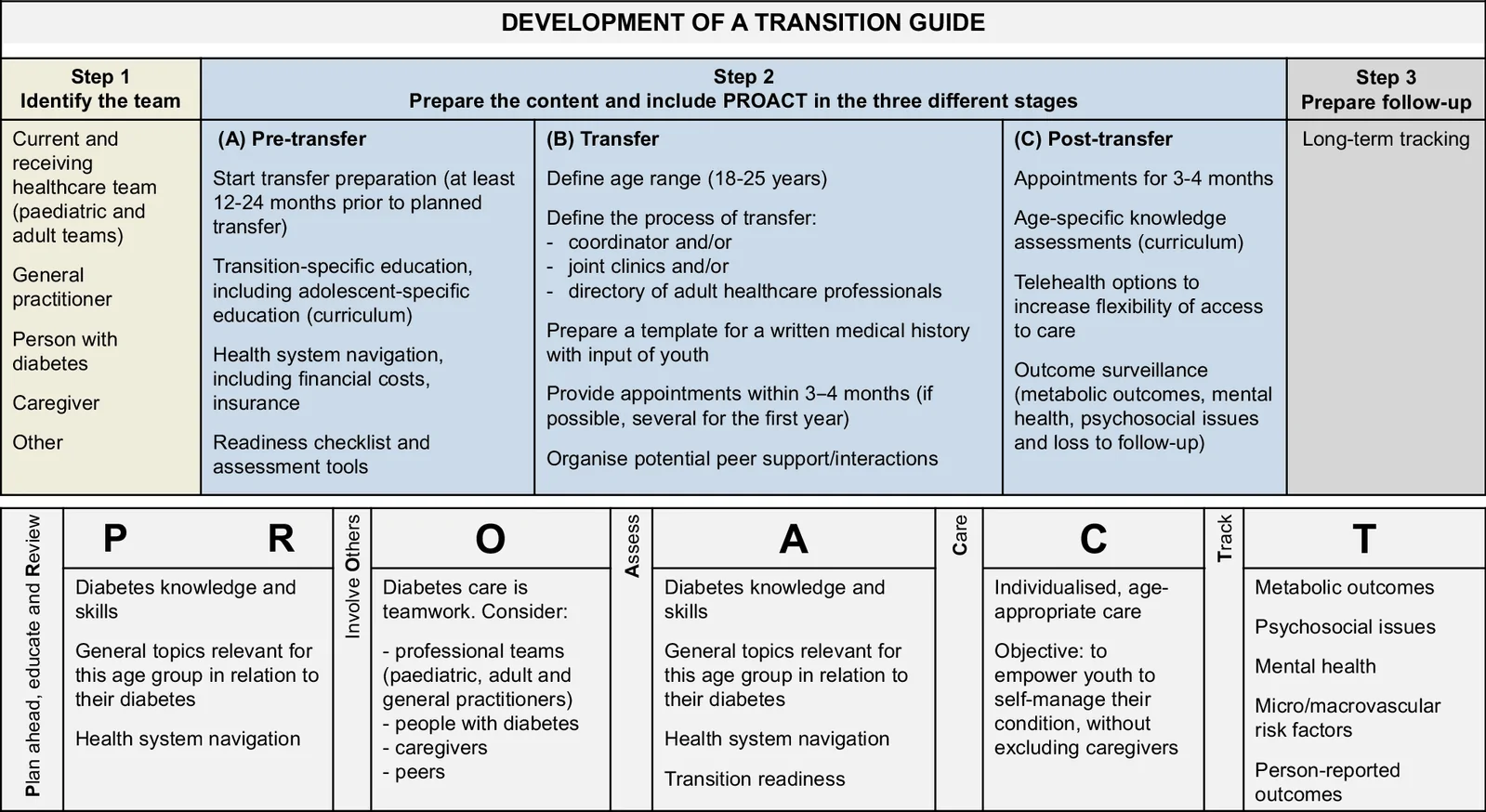
General principles and steps involved in developing a transition policy or guide

The inclusion of these strategies outlined in the consensus statements may occur at different stages in different locations. The team of healthcare professionals involved in the transfer of care may also differ in different sites. Figure 3 details the general principles and essential steps in formulating a related transition policy/guide.

### General transition consensus statements (1–11)

Diabetes self-management is a continuous activity for people with diabetes and their caregivers. People with diabetes face many challenges in young adulthood, which are not only diabetes-related, but also specific to their developmental phase (psychosocial, educational and vocational). These challenges can influence their diabetes self-management, interactions with healthcare professionals and, subsequently, the risk of acute and long-term diabetes-related complications.

The cornerstones of healthcare are education and empowerment, navigation of healthcare systems in both difficult and easy times, and the promotion of diabetes self-management. Diabetes care should be individualised and developmentally appropriate. Education should include general topics such as birth control, pregnancy planning and risks, and alcohol consumption and use of illicit products and their impact on glucose metabolism. The involvement of caregivers should be encouraged, but it is essential to prepare for the gradual transfer of responsibility to the person with diabetes. Healthcare professionals should empower young people. They should be aware of young people’s vulnerability and support them in the best possible way and, if needed, provide them with access to professionals with expertise and resources that may be of benefit. These resources may include online programmes, educational materials, and professional or peer support. At diagnosis and during routine follow-up care, screening for micro- and macrovascular risk factors and for psychosocial concerns, family factors and behavioural health concerns should be routinely performed, using validated tools where applicable. Appropriate management of these factors should occur when indicated.

### Pre-transfer consensus statements (12–18)

The first step for a smooth transition of care is the development of a transition policy/guide. This document should describe a centre’s/clinic’s approach to transition preparation (pre-transfer) and transfer to adult healthcare. The tasks and responsibilities of the different paediatric healthcare professionals in the transition process should be documented; local situations, team composition and healthcare systems will dictate how these roles are assigned. Similarly, the targeted age range for transfer should also be documented. Transition-related topics should be progressively integrated into the care provided. This should be initiated in a timely manner (consensus was obtained for initiation at least 12–24 months before the actual transfer). Education on transition-related knowledge and skills should be personalised with regular assessment of such knowledge and skills. The use of transition readiness assessment tools is helpful to identify potential gaps that need further discussion. The process should be transparent and interactive, with ongoing involvement of and exchange with the person with diabetes and their caregivers.

### Transfer consensus statements (19–26)

As conclusive long-term outcome data (metabolic and person reported) on the transfer from paediatric to adult diabetes care are lacking, different strategies for the transfer phase remain possible, dependent on the local situation. For the actual transfer, different approaches can be proposed, such as pre-planned appointments with the adult healthcare team, the use of a dedicated transition coordinator, joint clinics with paediatric and adult healthcare professionals, or peer support. Healthcare teams should be prepared to discuss possible insurance issues, financial costs or access to medicine. The development of a directory of adult care providers may be helpful. Regardless, communication between healthcare teams is important. In agreement with the person with diabetes, a written medical summary should be prepared and provided to the adult healthcare team. Ongoing education on transfer to adult care remains relevant.

### Post-transfer consensus statements (27–31)

Post-transfer, regular visits (every 3 months initially) with adult healthcare professionals should be scheduled, to evaluate diabetes self-management, integration in the changed healthcare setting, potential knowledge gaps and needs, and psychosocial health. Alternatives to traditional visits, such as telehealth and virtual care, can be offered in addition to improve communication with healthcare professionals and provide flexibility of access to care for people with diabetes with high levels of digital literacy.

## Discussion

To develop this consensus report, a group of experts with different professional backgrounds and including people with lived experience critically reviewed existing professional recommendations, based on evidence and expert opinion. In addition, they performed a systematic review (quantitative, qualitative), supplemented by international surveys capturing the views of people with diabetes, their caregivers and healthcare professionals, making this the most complete and up-to-date consensus report on the transition of youth with diabetes from paediatric to adult diabetes care services.

Consensus within the working group was obtained for 31 statements on the transition of care continuum and the three unique stages of pre-transfer, transfer and post-transfer for the transition from paediatric to adult diabetes care. After achievement of over 90% endorsement by the Delphi panel for the 26 statements with limited evidence (grade E), all statements were transformed into transition guidance with links to existing readiness and assessment tools [31, 33, 65–67]. As differences in healthcare systems and clinical and ambulatory care, as well as inter-individual differences, will influence the transition of care trajectory, this consensus report should be adjusted to local settings. Paediatric diabetes teams will have different compositions in different settings. In some centres the healthcare professional team will include specialised paediatricians, nurses, nurse educators, psychologists, social workers, dietitians and pharmacists, whereas in other centres it may include one general paediatrician and/or a nurse. It is important to define responsibilities with respect to transition. The template for the development and implementation of a diabetes-related transition policy/guide is universal, but the content should be specific to and feasible for the location where care will be provided. For example, recognising the vulnerability of people with diabetes and the provision of ongoing medical, mental and psychosocial support to empower them are vital. If a directory of adult healthcare professionals is not available at the time of transfer, alternative solutions could be identified, such as the use of peer support. This will not replace follow-up by adult healthcare professionals, but it may prevent people with diabetes dropping out of care. In collaboration with the scientific societies and their members, the development of a global directory, accessible online, could be envisaged. Over time, a structured approach towards the transition process will, together with long-term surveillance, provide better insights into best practice and cost-effectiveness.

Regardless of local setting, the diabetes-related transition policy/guide should provide clear information for people living with diabetes and their caregivers on the approach to transition preparation, readiness assessment, and the actual transfer to adult diabetes healthcare. This document should be developed in collaboration between the paediatric and adult diabetes healthcare teams involved in the transition process, people living with diabetes and their caregivers and, where possible, other key stakeholders, including general practitioners. Such a policy/guide should be shared with people living with diabetes who are receiving care and their caregivers 12–24 months before the anticipated time of transfer.

A clear policy/guide will include many of the consensus statements. For example, paediatric (and where applicable adult) diabetes healthcare professionals should be knowledgeable about their roles and responsibilities around transition planning and preparation for people with diabetes and their caregivers. They should help provide knowledge and skills so that people with diabetes and their caregivers can recognise and understand their own unique needs, facilitating transition to a different diabetes healthcare setting. Transition-related topics should be integrated into ongoing diabetes education in an age-appropriate and developmentally appropriate way, again commencing early on at least 12–24 months before transfer. Introducing transition-related topics early allows ample time to assess the diabetes-related knowledge and skills of the person with diabetes and identify gaps, with additional education provided where warranted. The education provided should not be limited to diabetes-specific topics but should also include general topics such as birth control and pregnancy planning (where applicable), use of alcohol and illicit drugs, and smoking.

Based on the setting, information on and preparation around potential changes in access to diabetes-related technology and medicines, financial costs and insurance coverage should be covered. Clear communication about these practical aspects may enable informed planning and help to reduce anxiety. Screening for metabolic outcomes and both micro- and macrovascular complications should also be conducted, as recommended in international consensus guidelines [38, 43]. This should include assessment of mental health and psychosocial issues, with appropriate action undertaken should problems be identified.

Finally, independent of the location, paediatric and adult diabetes healthcare professionals working with people with diabetes and their caregivers should be aware of the vulnerabilities of and different challenges experienced by those under their care, both diabetes- and non-diabetes-related. The support provided should be individualised, and treatment adherence and diabetes self-management should be encouraged. At all times it is important that people with diabetes and their caregivers feel comfortable voicing their transition-related questions and concerns. Timely and comprehensive communication between paediatric and adult diabetes healthcare teams is essential throughout. Key information, including the diabetes management and complication history of the people with diabetes, should be promptly disseminated. This communication helps to ensure clarity and alignment across diabetes healthcare teams, while emphasising patient-centred care. The use of a transition readiness checklist can help to ensure that comprehensive education and assessment is completed prior to transfer; multiple tools have been validated and are freely accessible [67]. Individual centres should decide which transition readiness tool they will use.

Tracking and monitoring the transition process and related outcomes will be essential to evaluate the transition process and quality of care provided and received. When adult and paediatric centres use electronic health records, ongoing evaluation and improvement will be more feasible. For example, the Plan Do Study Act (PDSA) cycle is a helpful tool for demonstrating value-based healthcare [68, 69]. Increased access to outcome data would allow implementation of this tool, generating the crucial robust and long-term data (at least 3–5 years) that are needed for better understanding of the transition process and ongoing quality improvement.

Although we fully acknowledge the potential risk of bias in the reported systematic reviews, the key outcome of these literature reviews is the pressing need for additional evidence, including longer term follow-up, consistent use of outcome indicators, studies not limited to analyses of metabolic control or access to emergency services, and better descriptions of determinants of successful transition (including ethnicity and gender) and critical characteristics of healthcare systems. They also highlight, in particular, the need for RCTs and longitudinal research. A particular gap is the lack of research on the transition of care in youth with type 2 diabetes, including the impact of healthcare system-related factors.

The development of a centre-based transition policy, taking into account the consensus statements and tracking/monitoring, is an essential step to improve the outcomes of transition.

As more evidence becomes available, there will be a need to update these consensus strategies.

In time, they may evolve into regular chapters in key publications, including the ISPAD clinical practice guidelines and ADA standards of care.

## Supplementary Information

Below is the link to the electronic supplementary material.

ESM (PDF 1.43 MB)

## Data Availability

The data that support the systematic review and Delphi survey are available on request. Data that support the perceptions and experiences of healthcare professionals and of people with diabetes and their caregivers are not available based on ethical approval constraints.
