# Supplementary material for: The transition of youth with diabetes from paediatric to adult diabetes care services: a consensus report of the joint working group of the International Society for Pediatric and Adolescent Diabetes (ISPAD), the European Association for the Study of Diabetes (EASD) and the American Diabetes Association (ADA)
Source: Diabetologia. 2026 Sep 14;69(12):3396–408. doi: 10.1007/s00125-026-06803-5 (PMC13714030; doi:10.1007/s00125-026-06803-5)
Supplement: Supplementary file 1 [file 125_2026_6803_MOESM1_ESM.pdf]

ESM 1: ACCORD checklist

Item No.	Section	Checklist Item (*help text*)	Page No.
T1	**Title**	Identify the article as reporting a consensus exercise and state the consensus methods used in the title.<br>*For example, Delphi or nominal group technique.*	The inclusion of the different methods in the title (consensus report of…., based on systemic and scoping reviews, combined with a Delphi procedure) was discussed within the group. The group proposes not to include it in the title, but it is clearly described in the methodology, in the abstract and the paper.
I1	**Introduction**	Explain why a consensus exercise was chosen over other approaches.	Background and Rationale<br>*Given the heterogeneity of diabetes care models ….*
I2		State the aim of the consensus exercise, including its intended audience and geographical scope (national, regional, global).	*Collectively this led …. Worldwide, were incorporated*
I3		If the consensus exercise is an update of an existing document, state why an update is needed, and provide the citation for the original document.	*As a starting point……..*
M1	**Methods Registration**	If the study or study protocol was prospectively registered, state the registration platform and provide a link. If the exercise was not registered, this should be stated.<br>*Recommended to include the date of registration.*	This work was started before the ACCORD paper was published.
M2		Describe the role(s) and areas of expertise or experience of those directing the consensus exercise.	The 3 participating societies each identified 3 members with work experience and/or scientific knowledge; each renowned in the

	**Selection of SC and/or panellists**	*For example, whether the project was led by a chair, co-chairs or a steering committee, and, if so, how they were chosen. List their names if appropriate, and whether there were any subgroups for individual steps in the process.*	field, of different professional and geographical backgrounds and/or lived experience.<br>ISPAD: A Gomber (early career, public health and person with diabetes, India), S James (diabetes nurse educator, person with diabetes, Australia) and SM Ng (paediatric diabetologist, UK); ADA: N Maruthur (general practitioner. USA), J Weissberg-Benchell (psychologist USA), and S Lyons (paediatric diabetologist USA); and EASD: E Hagstrom (adult diabetologist, Sweden), A Araszkiewicz (adult diabetologist, Poland) and F Snoek (psychologist, The Netherlands). The chair is a member of the 3 societies and initiated the work (C de Beaufort, paediatric diabetologist, Luxembourg). Finally, an early career person of ISPAD was invited to include the next generation professionals (L Cudizio, paediatric diabetologist, Brazil). J Weissberg-Benchell led the systematic review, S James the scoping review for health care professionals and L Cudizio the scoping review for people with diabetes and their carers.
M3		Explain the criteria for panellist inclusion and the rationale for panellist numbers. State who was responsible for panellist selection.	The number of participants in the working group was determined by the 3 societies, and represented diversity in profession, and geographical location. For the Delphi panel composition details are provided in the text, *Delphi survey.*

M4		Describe the recruitment process (how panellists were invited to participate).<br>*Include communication/advertisement method(s) and locations, numbers of invitations sent, and whether there was centralised oversight of invitations or if panellists were asked/allowed to suggest other members of the panel.*	See above, *Delphi survey.*
M5		Describe the role of any members of the public, patients or carers in the different steps of the study.	All were involved in all steps of the study/work.
M6	**Preparatory research**	Describe how information was obtained prior to generating items or other materials used during the consensus exercise.<br>*This might include a literature review, interviews, surveys, or another process.*	Previous Cochrane review (2016 doi: 10.1002/14651858.CD009794.pub2.) and position statement (2011 doi: 10.2337/dc11-1723), documents in the guidelines of the 3 societies combined society statements and clinical care consensus guidelines (ADA Standards of care, and ISPAD Clinical practice guidelines).
M7		Describe any systematic literature search in detail, including the search strategy and dates of search or the citation if published already.<br>*Provide the details suggested by the reporting guideline PRISMA and the related PRISMA-Search extension.*	Systematic review approach is reported in the Methodology section, and in the ESM 3/4<br>Published papers<br>doi: 10.1007/s11892-025-01578-2<br>doi.org/10.2337/cd25-0048
M8		Describe how any existing scientific evidence was summarised and if this evidence was provided to the panellists.	The working group identified and discussed existing documents, and the new data generated through the reviews. Based on these, the 31 statements were formulated. Those with evidence A/B

			were included but not submitted for consensus (see M9). For the remaining 26, a pilot was run. After minor edits of the wording, approved unanimously by the full working group, the 26 statements were submitted to the Delphi panel (see M9).
M9	**Assessing consensus**	Describe the methods used and steps taken to gather panellist input and reach consensus (for example, Delphi, RAND-UCLA, nominal group technique).<br>*If modifications were made to the method in its original form, provide a detailed explanation of how the method was adjusted and why this was necessary for the purpose of your consensus-based study.*	The Delphi panel was generated with the input of the 3 societies, considering geodiversity and professional diversity, with the objective being to establish consensus among the target population (health care professionals from paediatric, adult and general practice) and the online Diabetes community (#DeDoc). As summarized, 69,5 % of those invited accepted the invitation, shared their professional background and received the survey. To optimize feedback, the survey was mailed on three occasions, with fully completed surveys received by n=44 of the 57 participants. Data collection was anonymous in nature, undertaken using Qualtrics software. All items met the preset threshold of >70% with strongly agree/agree >90 % but when including in the denominator the 57 the outcome > 70 % was still met.
M10		Describe how each question or statement was presented and the response options. State whether panellists were able to or required to explain their responses, and whether they could propose new items.	All the statements are endorsed and are included in Table 2.<br>Open text allowed comments, which were evaluated by the group<br>*(Where panellist had provided comments….*

		Where possible, present the questionnaire or list of statements as supplementary material.	
M11		State the objective of each consensus step.<br>*A step could be a consensus meeting, a discussion or interview session, or a Delphi round.*	Results include the different steps, systematic reviews, and surveys. Once the pilot and validation of the statements were completed by a small panel or working group, the statements were presented to a larger group to include diverse professionals and individuals/carers with diabetes. This group endorsed all items (preset>70%) The panel was recognized for their input by mail, with no further exchange.
M12		State the definition of consensus (for example, number, percentage, or categorical rating, such as 'agree' or 'strongly agree') and explain the rationale for that definition.	See above: M9.
M13		State whether items that met the prespecified definition of consensus were included in any subsequent voting rounds.	Not applicable.
M14		For each step, describe how responses were collected, and whether responses were collected in a group setting or individually.	Qualtrics software, anonymously with options for comments.
M15		Describe how responses were processed and/or synthesised. *Include qualitative analyses of free-text responses (for example, thematic, content or cluster analysis) and/or quantitative analytical methods, if used.*	Direct analysis in 4 subgroups on the 4 stages of transition: continuum, pre-transfer, transfer and post transfer. All were shared and discussed in the working group.

M16		Describe any piloting of the study materials and/or survey instruments.<br>*Include how many individuals piloted the study materials, the rationale for the selection of those individuals, any changes made as a result and whether their responses were used in the calculation of the final consensus. If no pilot was conducted, this should be stated.*	Working group piloted.
M17		If applicable, describe how feedback was provided to panellists at the end of each consensus step or meeting.<br>*State whether feedback was quantitative (for example, approval rates per topic/item) and/or qualitative (for example, comments, or lists of approved items), and whether it was anonymised.*	After the Delphi procedure and endorsement, all participants received an email detailing the outcome.
M18		State whether anonymity was planned in the study design. Explain where and to whom it was applied and what methods were used to guarantee anonymity.	Pilot by the working group was not anonymous. The Delphi process was anonymous, based on the technical approach (Qualtrics software). This has as limitations that we do not know which panellists have not replied.
M19		State if the steering committee was involved in the decisions made by the consensus panel.<br>*For example, whether the steering committee or those managing consensus also had voting rights.*	N/A.

M20	**Participation**	Describe any incentives used to encourage responses or participation in the consensus process.<br>*For example, were invitations to participate reiterated, or were participants reimbursed for their time.*	Invitations were sent out twice to the largest group. For those who replied and accepted, the survey was shared on three occasions.
M21		Describe any adaptations to make the surveys/meetings more accessible.<br>*For example, the languages in which the surveys/meetings were conducted and whether translations or plain language summaries were available*.	N/A.
R1	**Results**	State when the consensus exercise was conducted. List the date of initiation and the time taken to complete each consensus step, analysis, and any extensions or delays in the analysis.	Start of working group 2023.<br>Systematic review/Scoping reviews undertaken 2023-2024, with publications 2024-2025.<br>Submission of the consensus to the society boards 2025.
R2		Explain any deviations from the study protocol, and why these were necessary.<br>*For example, addition of panel members during the exercise, number of consensus steps, stopping criteria; report the step(s) in which this occurred.*	No changes, but initial planning was 12 months. Due to the decision to run a systematic review and 2 surveys, the timeline was extended.
R3		For each step, report quantitative (number of panellists, response rate) and qualitative (relevant socio-demographics) data to describe the participating panellists.	Included in the text and table 1.

R4		Report the final outcome of the consensus process as qualitative (for example, aggregated themes from comments) and/or quantitative (for example, summary statistics, score means, medians and/or ranges) data.	Included in the text: the outcome is the list of endorsed statements and table 2
R5		List any items or topics that were modified or removed during the consensus process. Include why and when in the process they were modified or removed.	Modification of 'healthcare provider' to 'healthcare professional'.
D1	**Discussion**	Discuss the methodological strengths and limitations of the consensus exercise.<br>*Include factors that may have impacted the decisions (for example, response rates, representativeness of the panel, potential for feedback during consensus to bias responses, potential impact of any non-anonymised interactions).*	The methodological strength is the inclusion of the target population in all steps (steering/Delphi and the different survey). The Delphi survey was anonymous and although the composition of the panel, those who accepted to participate, was known, we do not know the professional background of those who answered.
D2		Discuss whether the recommendations are consistent with any pre-existing literature and, if not, propose reasons why this process may have arrived at alternative conclusions.	The recommendations are overall consistent with pre existing literature.
O1	**Other information**	List any endorsing organisations involved and their role.	Three partnering societies and online community for the Delphi panel.
O2		State any potential conflicts of interests, including among those directing the consensus study and panellists. Describe how conflicts of interest were managed.	No conflict of interest.
O3		State any funding received and the role of the funder.	Included.

		Specify, for example, any funder involvement in the study concept/design, participation in the steering committee, conducting the consensus process, funding of any medical writing support. This could be disclosed in the methods or in the relevant transparency section of the manuscript. Where a funder did not play a role in the process or influence the decisions reached, this should be specified.	

From: PLoS Med 21(1): e1004326. https://doi.org/10.1371/journal.pmed.1004326 For more information see: https://www.ismpp.org/accord

AMSTAR 2: a critical appraisal tool for systematic reviews that include randomised or non-randomised studies of healthcare interventions, or both

ESM 2 : AGREE checklist

AGREE Reporting Checklist

2016

This checklist is intended to guide the reporting of clinical practice guidelines.

CHECKLIST ITEM AND DESCRIPTION	REPORTING CRITERIA	Page # (Sections)
DOMAIN 1: SCOPE AND PURPOSE		
**1. OBJECTIVES**<br>*Report the overall objective(s) of the guideline. The expected health benefits from the guideline are to be specific to the clinical problem or health topic.*	☒ Health intent(s) (i.e., prevention, screening, diagnosis, treatment, etc.)<br>☒ Expected benefit(s) or outcome(s)<br>☒ Target(s) (e.g., patient population, society)	Background and Rationale<br>Results
**2. QUESTIONS**<br>*Report the health question(s) covered by the guideline, particularly for the key recommendations.*	☒ Target population<br>☒ Intervention(s) or exposure(s)<br>☐ Comparisons (if appropriate)<br>☒ Outcome(s)<br>☒ Health care setting or context	Background and Rationale<br>Methodology<br>Results
**3. POPULATION**<br>*Describe the population (i.e., patients, public, etc.) to whom the guideline is meant to apply.*	☒ Target population, sex and age<br>☒ Clinical condition (if relevant)<br>☒ Severity/stage of disease (if relevant)<br>☒ Comorbidities (if relevant)<br>☐ Excluded populations (if relevant)	Background and Rationale
DOMAIN 2: STAKEHOLDER INVOLVEMENT		
**4. GROUP MEMBERSHIP**<br>*Report all individuals who were involved in the development process. This may include members of the steering group, the research team involved in selecting and reviewing/rating the evidence and individuals involved in formulating the final recommendations.*	☒ Name of participant<br>☒ Discipline/content expertise (e.g., neurosurgeon, methodologist)<br>☒ Institution (e.g., St. Peter's hospital)<br>☒ Geographical location (e.g., Seattle, WA)<br>☒ A description of the member's role in the guideline development group	Authorship,<br>Methodology<br>Delphi survey<br>Discussion
**5. TARGET POPULATION PREFERENCES AND VIEWS**<br>*Report how the views and preferences of the target population were sought/considered and what the resulting outcomes were.*	☒ Statement of type of strategy used to capture patients'/publics' views and preferences (e.g., participation in the guideline development group, literature review of values and preferences)<br>☒ Methods by which preferences and views were sought (e.g., evidence from literature, surveys, focus groups)<br>☒ Outcomes/information gathered on patient/public information<br>☒ How the information gathered was used to inform the guideline development process and/or formation of the recommendations	Methodology,<br>Results
**6. TARGET USERS**	☒ The intended guideline audience (e.g.	Background and Rationale

Report the target (or intended) users of the guideline.	specialists, family physicians, patients, clinical or institutional leaders/administrators)<br>☒ How the guideline may be used by its target audience (e.g., to inform clinical decisions, to inform policy, to inform standards of care)	Discussion
DOMAIN 3: RIGOUR OF DEVELOPMENT		
**7. SEARCH METHODS**<br>*Report details of the strategy used to search for evidence.*	☒ Named electronic database(s) or evidence source(s) where the search was performed (e.g., MEDLINE, EMBASE, PsychINFO, CINAHL)<br>☒ Time periods searched (e.g., January 1, 2004 to March 31, 2008)<br>☒ Search terms used (e.g., text words, indexing terms, subheadings)<br>☒ Full search strategy included (e.g., possibly located in appendix)	Methodology
**8. EVIDENCE SELECTION CRITERIA**<br>*Report the criteria used to select (i.e., include and exclude) the evidence. Provide rationale, where appropriate.*	☒ Target population (patient, public, etc.) characteristics<br>☒ Study design<br>☒ Comparisons (if relevant)<br>☒ Outcomes<br>☒ Language (if relevant)<br>☐ Context (if relevant)	Methodology
**9. STRENGTHS & LIMITATIONS OF THE EVIDENCE**<br>*Describe the strengths and limitations of the evidence. Consider from the perspective of the individual studies and the body of evidence aggregated across all the studies. Tools exist that can facilitate the reporting of this concept.*	☒ Study design(s) included in body of evidence<br>☒ Study methodology limitations (sampling, blinding, allocation concealment, analytical methods)<br>☒ Appropriateness/relevance of primary and secondary outcomes considered<br>☒ Consistency of results across studies<br>☒ Direction of results across studies<br>☐ Magnitude of benefit versus magnitude of harm<br>☒ Applicability to practice context	Methodology
**10. FORMULATION OF RECOMMENDATIONS**<br>*Describe the methods used to formulate the recommendations and how final decisions were reached. Specify any areas of disagreement and the methods used to resolve them.*	☒ Recommendation development process (e.g., steps used in modified Delphi technique, voting procedures that were considered)<br>☒ Outcomes of the recommendation development process (e.g., extent to which consensus was reached using modified Delphi technique, outcome of voting procedures)<br>☒ How the process influenced the recommendations (e.g., results of Delphi technique influence final recommendation, alignment with recommendations and the final vote)	Methodology<br>Delphi Survey
**11. CONSIDERATION OF BENEFITS AND HARMS**<br>*Report the health benefits, side effects, and risks that were considered when*	☒ Supporting data and report of benefits<br>☒ Supporting data and report of harms/side effects/risks<br>☐ Reporting of the balance/trade-off between	Discussion

formulating the recommendations.	benefits and harms/side effects/risks<br>☐ Recommendations reflect considerations of both benefits and harms/side effects/risks	
**12. LINK BETWEEN RECOMMENDATIONS AND EVIDENCE**<br>*Describe the explicit link between the recommendations and the evidence on which they are based.*	☒ How the guideline development group linked and used the evidence to inform recommendations<br>☒ Link between each recommendation and key evidence (text description and/or reference list)<br>☒ Link between recommendations and evidence summaries and/or evidence tables in the results section of the guideline	Results Discussion, No action is a known risk
**13. EXTERNAL REVIEW**<br>*Report the methodology used to conduct the external review.*	☒ Purpose and intent of the external review (e.g., to improve quality, gather feedback on draft recommendations, assess applicability and feasibility, disseminate evidence)<br>☐ Methods taken to undertake the external review (e.g., rating scale, open-ended questions)<br>☐ Description of the external reviewers (e.g., number, type of reviewers, affiliations)<br>☐ Outcomes/information gathered from the external review (e.g., summary of key findings)<br>☐ How the information gathered was used to inform the guideline development process and/or formation of the recommendations (e.g., guideline panel considered results of review in forming final recommendations)	Peer reviews through Diabetologia and external reviews through ISPAD, EASD and ADA
**14. UPDATING PROCEDURE**<br>*Describe the procedure for updating the guideline.*	☒ A statement that the guideline will be updated<br>☐ Explicit time interval or explicit criteria to guide decisions about when an update will occur<br>☐ Methodology for the updating procedure	Discussion
DOMAIN 4: CLARITY OF PRESENTATION		
**15. SPECIFIC AND UNAMBIGUOUS RECOMMENDATIONS**<br>*Describe which options are appropriate in which situations and in which population groups, as informed by the body of evidence.*	☒ A statement of the recommended action<br>☒ Intent or purpose of the recommended action (e.g., to improve quality of life, to decrease side effects)<br>☒ Relevant population (e.g., patients, public)<br>☐ Caveats or qualifying statements, if relevant (e.g., patients or conditions for whom the recommendations would not apply)<br>☒ If there is uncertainty about the best care option(s), the uncertainty should be stated in the guideline	Results, figure 2, figure 3 Discussion
**16. MANAGEMENT OPTIONS**<br>*Describe the different options for managing the condition or health issue.*	☒ Description of management options<br>☐ Population or clinical situation most appropriate to each option	Figure 2 and figure 3
**17. IDENTIFIABLE KEY RECOMMENDATIONS**<br>*Present the key recommendations so that*	☒ Recommendations in a summarized box, typed in bold, underlined, or presented as flow charts or algorithms	Results Figure 2 and Figure 3

they are easy to identify.	☒ Specific recommendations grouped together in one section	
DOMAIN 5: APPLICABILITY		
**18. FACILITATORS AND BARRIERS TO APPLICATION**<br>*Describe the facilitators and barriers to the guideline's application.*	☒ Types of facilitators and barriers that were considered<br>☒ Methods by which information regarding the facilitators and barriers to implementing recommendations were sought (e.g., feedback from key stakeholders, pilot testing of guidelines before widespread implementation)<br>☐ Information/description of the types of facilitators and barriers that emerged from the inquiry (e.g., practitioners have the skills to deliver the recommended care, sufficient equipment is not available to ensure all eligible members of the population receive mammography)<br>☒ How the information influenced the guideline development process and/or formation of the recommendations	Methodology delphi survey
**19. IMPLEMENTATION ADVICE/TOOLS**<br>*Provide advice and/or tools on how the recommendations can be applied in practice.*	☒ Additional materials to support the implementation of the guideline in practice. For example:<br>• Guideline summary documents<br>• Links to check lists, algorithms<br>• Links to how-to manuals<br>○ Solutions linked to barrier analysis (see Item 18)<br>○ Tools to capitalize on guideline facilitators (see Item 18)<br>○ Outcome of pilot test and lessons learned	Figure 2 and figure 3
**20. RESOURCE IMPLICATIONS**<br>*Describe any potential resource implications of applying the recommendations.*	☐ Types of cost information that were considered (e.g., economic evaluations, drug acquisition costs)<br>☐ Methods by which the cost information was sought (e.g., a health economist was part of the guideline development panel, use of health technology assessments for specific drugs, etc.)<br>☐ Information/description of the cost information that emerged from the inquiry (e.g., specific drug acquisition costs per treatment course)<br>☒ How the information gathered was used to inform the guideline development process and/or formation of the recommendations	Scientific publications, reporting on health care Utilization
**21. MONITORING/ AUDITING CRITERIA**<br>*Provide monitoring and/or auditing criteria to measure the application of guideline recommendations.*	☒ Criteria to assess guideline implementation or adherence to recommendations<br>☒ Criteria for assessing impact of implementing the recommendations<br>☒ Advice on the frequency and interval of	Figure 2 and figure 3

	measurement<br>☐ Operational definitions of how the criteria should be measured	
DOMAIN 6: EDITORIAL INDEPENDENCE		
**22. FUNDING BODY**<br>*Report the funding body's influence on the content of the guideline.*	☒ The name of the funding body or source of funding (or explicit statement of no funding)<br>☒ A statement that the funding body did not influence the content of the guideline	Funding
**23. COMPETING INTERESTS**<br>*Provide an explicit statement that all group members have declared whether they have any competing interests.*	☒ Types of competing interests considered<br>☒ Methods by which potential competing interests were sought<br>☒ A description of the competing interests<br>☐ How the competing interests influenced the guideline process and development of recommendations	Authors relationships and activities

From:
Brouwers MC, Kerkvliet K, Spithoff K, on behalf of the AGREE Next Steps Consortium. The AGREE Reporting Checklist: a tool to improve reporting of clinical practice guidelines. *BMJ* 2016;352:i1152. doi: 10.1136/bmj.i1152.

For more information about the AGREE Reporting Checklist, please visit the AGREE Enterprise website at http://www.agreetrust.org.

AMSTAR 2: a critical appraisal tool for systematic reviews that include randomised or non-randomised studies of healthcare interventions, or both

1. Did the research questions and inclusion criteria for the review include the components of PICO?

For Yes:	Optional (recommended)	
☒ Population	☐ Timeframe for follow-up	☒ Yes
☒ Intervention		☐ No
☒ Comparator group		
☒ Outcome		

2. Did the report of the review contain an explicit statement that the review methods were established prior to the conduct of the review and did the report justify any significant deviations from the protocol?

For Partial Yes: The authors state that they had a written protocol or guide that included ALL the following:	For Yes: As for partial yes, plus the protocol should be registered and should also have specified:	
		☐ Yes
☒ review question(s)	☐ a meta-analysis/synthesis plan, if appropriate, *and*	☒ Partial Yes
☒ a search strategy	☐ a plan for investigating causes of heterogeneity	☐ No
☒ inclusion/exclusion criteria	☐ justification for any deviations from the protocol	
☒ a risk of bias assessment		

3. Did the review authors explain their selection of the study designs for inclusion in the review?

For Yes, the review should satisfy ONE of the following:	
☐ *Explanation for* including only RCTs	☒ Yes
☐ OR *Explanation for* including only NRSI	☐ No
☒ OR *Explanation for* including both RCTs and NRSI	

4. Did the review authors use a comprehensive literature search strategy?

For Partial Yes (all the following):	For Yes, should also have (all the following):	
☒ searched at least 2 databases (relevant to research question)	☐ searched the reference lists / bibliographies of included studies	☐ Yes
☒ provided key word and/or search strategy	☐ searched trial/study registries	☒ Partial Yes
☒ justified publication restrictions (e.g. language)	☐ included/consulted content experts in the field	☐ No
	☐ where relevant, searched for grey literature	
	☒ conducted search within 24 months of completion of the review	

5. Did the review authors perform study selection in duplicate?

For Yes, either ONE of the following:	
☒ at least two reviewers independently agreed on selection of eligible studies and achieved consensus on which studies to include	☒ Yes
☐ OR two reviewers selected a sample of eligible studies and achieved good agreement (at least 80 percent), with the remainder selected by one reviewer.	☐ No

6. Did the review authors perform data extraction in duplicate?

For Yes, either ONE of the following:

- ☒ at least two reviewers achieved consensus on which data to extract from included studies
- ☐ OR two reviewers extracted data from a sample of eligible studies and achieved good agreement (at least 80 percent), with the remainder extracted by one reviewer.

☒ Yes
☐ No

7. Did the review authors provide a list of excluded studies and justify the exclusions?

For Partial Yes:

- ☐ provided a list of all potentially relevant studies that were read in full-text form but excluded from the review

For Yes, must also have:

- ☐ Justified the exclusion from the review of each potentially relevant study

☐ Yes
☐ Partial Yes
☒ No

The justification for exclusion is defined in the design and numbers are shown in the PRISMA figure the studies themselves are not further detailed

8. Did the review authors describe the included studies in adequate detail?

For Partial Yes (ALL the following):

- ☒ described populations
- ☒ described interventions
- ☒ described comparators
- ☒ described outcomes
- ☒ described research designs

For Yes, should also have ALL the following:

- ☒ described population in detail
- ☒ described intervention in detail (including doses where relevant)
- ☒ described comparator in detail (including doses where relevant)
- ☒ described study's setting
- ☒ timeframe for follow-up

☒ Yes
☐ Partial Yes
☐ No

9. Did the review authors use a satisfactory technique for assessing the risk of bias (RoB) in individual studies that were included in the review?

For assessing quality of the studies , the JBI checklists have been applied and results of these are included under S1 in the paper .

RCTs

For Partial Yes, must have assessed RoB from

- ☐ unconcealed allocation, *and*
- ☐ lack of blinding of patients and assessors when assessing outcomes (unnecessary for objective outcomes such as all-cause mortality)

For Yes, must also have assessed RoB from:

- ☐ allocation sequence that was not truly random, *and*
- ☐ selection of the reported result from among multiple measurements or analyses of a specified outcome

☐ Yes
☒ Partial Yes
☐ No
☐ Includes only NRSI

NRSI

For Partial Yes, must have assessed RoB:

- ☒ from confounding, *and*
- ☒ from selection bias

For Yes, must also have assessed RoB:

- ☐ methods used to ascertain exposures and outcomes, *and*
- ☐ selection of the reported result from among multiple measurements or analyses of a specified outcome

☐ Yes
☒ Partial Yes
☐ No
☐ Includes only RCTs

10. Did the review authors report on the sources of funding for the studies included in the review?

For Yes

- ☐ Must have reported on the sources of funding for individual studies included in the review. Note: Reporting that the reviewers looked for this information but it was not reported by study authors also qualifies

☐ Yes
☒ No

11. If meta-analysis was performed did the review authors use appropriate methods for statistical combination of results?

RCTs
For Yes:

- ☐ The authors justified combining the data in a meta-analysis
  - ☐ AND they used an appropriate weighted technique to combine study results and adjusted for heterogeneity if present.
  - ☐ AND investigated the causes of any heterogeneity

☐ Yes
☐ No
☒ No meta-analysis conducted

For NRSI
For Yes:

- ☐ The authors justified combining the data in a meta-analysis
  - ☐ AND they used an appropriate weighted technique to combine study results, adjusting for heterogeneity if present
  - ☐ AND they statistically combined effect estimates from NRSI that were adjusted for confounding, rather than combining raw data, or justified combining raw data when adjusted effect estimates were not available
  - ☐ AND they reported separate summary estimates for RCTs and NRSI separately when both were included in the review

☐ Yes
☐ No
☒ No meta-analysis conducted

12. If meta-analysis was performed, did the review authors assess the potential impact of RoB in individual studies on the results of the meta-analysis or other evidence synthesis?

For Yes:

- ☐ included only low risk of bias RCTs
- ☐ OR, if the pooled estimate was based on RCTs and/or NRSI at variable RoB, the authors performed analyses to investigate possible impact of RoB on summary estimates of effect.

☐ Yes
☐ No
☒ No meta-analysis conducted

13. Did the review authors account for RoB in individual studies when interpreting/ discussing the results of the review?

For Yes:

- ☐ included only low risk of bias RCTs
- ☒ OR, if RCTs with moderate or high RoB, or NRSI were included the review provided a discussion of the likely impact of RoB on the results

☒ Yes
☐ No

14. Did the review authors provide a satisfactory explanation for, and discussion of, any heterogeneity observed in the results of the review?

For Yes:

- ☐ There was no significant heterogeneity in the results
- ☒ OR if heterogeneity was present the authors performed an investigation of sources of any heterogeneity in the results and discussed the impact of this on the results of the review

☒ Yes
☐ No

15. If they performed quantitative synthesis did the review authors carry out an adequate investigation of publication bias (small study bias) and discuss its likely impact on the results of the review?

For Yes:

- ☐ performed graphical or statistical tests for publication bias and discussed the likelihood and magnitude of impact of publication bias

☐ Yes
☐ No
☒ No meta-analysis conducted

16. Did the review authors report any potential sources of conflict of interest, including any funding they received for conducting the review?

For Yes:	
☒ The authors reported no competing interests OR	☒ Yes
☐ The authors described their funding sources and how they managed potential conflicts of interest	☐ No

To cite this tool: Shea BJ, Reeves BC, Wells G, Thuku M, Hamel C, Moran J, Moher D, Tugwell P, Welch V, Kristjansson E, Henry DA. AMSTAR 2: a critical appraisal tool for systematic reviews that include randomised or non-randomised studies of healthcare interventions, or both. BMJ. 2017 Sep 21;358:j4008.

x

ESM4

AMSTAR 2: a critical appraisal tool for systematic reviews that include randomised or non-randomised studies of healthcare interventions, or both

1. Did the research questions and inclusion criteria for the review include the components of PICO?

For Yes:
- [x] Population
- [ ] Intervention
- [ ] Comparator group
- [x] Outcome

Study designs are included

N/A, No RCT , non intervention non qualitative

Optional (recommended)
- [ ] Timeframe for follow-up

- [ ] Yes
- [ ] No

2. Did the report of the review contain an explicit statement that the review methods were established prior to the conduct of the review and did the report justify any significant deviations from the protocol?

For Partial Yes:
The authors state that they had a written protocol or guide that included ALL the following:
- [x] review question(s)
- [x] a search strategy
- [x] inclusion/exclusion criteria
- [x] a risk of bias assessment

For Yes:
As for partial yes, plus the protocol should be registered and should also have specified:
- [ ] a meta-analysis/synthesis plan, if appropriate, *and*
- [ ] a plan for investigating causes of heterogeneity
- [ ] justification for any deviations from the protocol

- [ ] Yes
- [x] Partial Yes
- [ ] No

3. Did the review authors explain their selection of the study designs for inclusion in the review?

For Yes, the review should satisfy ONE of the following:
- [ ] *Explanation for* including only RCTs
- [x] OR *Explanation for* including only NRSI
- [ ] OR *Explanation for* including both RCTs and NRSI

- [x] Yes
- [ ] No

4. Did the review authors use a comprehensive literature search strategy?

For Partial Yes (all the following):
- [x] searched at least 2 databases (relevant to research question)
- [x] provided key word and/or search strategy
- [x] justified publication restrictions (e.g. language)

For Yes, should also have (all the following):
- [ ] searched the reference lists / bibliographies of included studies
- [ ] searched trial/study registries
- [ ] included/consulted content experts in the field
- [ ] where relevant, searched for grey literature
- [x] conducted search within 24 months of completion of the review

- [ ] Yes
- [x] Partial Yes
- [ ] No

5. Did the review authors perform study selection in duplicate?

For Yes, either ONE of the following:
- [x] at least two reviewers independently agreed on selection of eligible studies and achieved consensus on which studies to include
- [ ] OR two reviewers selected a sample of eligible studies and achieved good agreement (at least 80 percent), with the remainder selected by one reviewer.

- [x] Yes
- [ ] No

6. Did the review authors perform data extraction in duplicate?

For Yes, either ONE of the following:

- [x] at least two reviewers achieved consensus on which data to extract from included studies
- [ ] OR two reviewers extracted data from a sample of eligible studies and achieved good agreement (at least 80 percent), with the remainder extracted by one reviewer.

- [x] Yes
- [ ] No

7. Did the review authors provide a list of excluded studies and justify the exclusions?

For Partial Yes:

- [ ] provided a list of all potentially relevant studies that were read in full-text form but excluded from the review

For Yes, must also have:

- [ ] Justified the exclusion from the review of each potentially relevant study

- [ ] Yes
- [ ] Partial Yes
- [x] No

The justification for exclusion is defined in the design and numbers are shown in the PRISMA figure the studies themselves are not further detailed

8. Did the review authors describe the included studies in adequate detail?

For Partial Yes (ALL the following):

- [x] described populations
- N/A described interventions
- N/A described comparators
- [x] described outcomes
- [x] described research designs

For Yes, should also have ALL the following:

- [x] described population in detail
- N/A described intervention in detail (including doses where relevant)
- N/A described comparator in detail (including doses where relevant)
- [x] described study's setting
- [x] timeframe for follow-up

- [x] Yes
- [ ] Partial Yes
- [ ] No

9. Did the review authors use a satisfactory technique for assessing the risk of bias (RoB) in individual studies that were included in the review?

For assessing the studies , the JBI checklists have been applied and results of these are included under S1 in the paper

RCTs

For Partial Yes, must have assessed RoB from

- [ ] unconcealed allocation, *and*
- [ ] lack of blinding of patients and assessors when assessing outcomes (unnecessary for objective outcomes such as all-cause mortality)

For Yes, must also have assessed RoB from:

- [ ] allocation sequence that was not truly random, *and*
- [ ] selection of the reported result from among multiple measurements or analyses of a specified outcome

- [ ] Yes
- [ ] Partial Yes
- [ ] No
- [x] Includes only NRSI

NRSI

For Partial Yes, must have assessed RoB:

- [x] from confounding, *and*
- [x] from selection bias

For Yes, must also have assessed RoB:

- [ ] methods used to ascertain exposures and outcomes, *and*
- [ ] selection of the reported result from among multiple measurements or analyses of a specified outcome

- [ ] Yes
- [x] Partial Yes
- [ ] No
- [ ] Includes only RCTs

10. Did the review authors report on the sources of funding for the studies included in the review?

For Yes

- [ ] Must have reported on the sources of funding for individual studies included in the review. Note: Reporting that the reviewers looked for this information but it was not reported by study authors also qualifies

- [ ] Yes
- [x] No

11. If meta-analysis was performed did the review authors use appropriate methods for statistical combination of results?

RCTs
For Yes:

- ☐ The authors justified combining the data in a meta-analysis
  - ☐ AND they used an appropriate weighted technique to combine study results and adjusted for heterogeneity if present.
  - ☐ AND investigated the causes of any heterogeneity

☐ Yes
☐ No
☒ No meta-analysis conducted

For NRSI
For Yes:

- ☐ The authors justified combining the data in a meta-analysis
  - ☐ AND they used an appropriate weighted technique to combine study results, adjusting for heterogeneity if present
  - ☐ AND they statistically combined effect estimates from NRSI that were adjusted for confounding, rather than combining raw data, or justified combining raw data when adjusted effect estimates were not available
  - ☐ AND they reported separate summary estimates for RCTs and NRSI separately when both were included in the review

☐ Yes
☐ No
☒ No meta-analysis conducted

12. If meta-analysis was performed, did the review authors assess the potential impact of RoB in individual studies on the results of the meta-analysis or other evidence synthesis?

For Yes:

- ☐ included only low risk of bias RCTs
- ☐ OR, if the pooled estimate was based on RCTs and/or NRSI at variable RoB, the authors performed analyses to investigate possible impact of RoB on summary estimates of effect.

☐ Yes
☐ No
☒ No meta-analysis conducted

13. Did the review authors account for RoB in individual studies when interpreting/ discussing the results of the review?

For Yes:

- ☐ included only low risk of bias RCTs
- ☒ OR, if RCTs with moderate or high RoB, or NRSI were included the review provided a discussion of the likely impact of RoB on the results

☒ Yes
☐ No

14. Did the review authors provide a satisfactory explanation for, and discussion of, any heterogeneity observed in the results of the review?

For Yes:

- ☐ There was no significant heterogeneity in the results
- ☒ OR if heterogeneity was present the authors performed an investigation of sources of any heterogeneity in the results and discussed the impact of this on the results of the review

☒ Yes
☐ No

15. If they performed quantitative synthesis did the review authors carry out an adequate investigation of publication bias (small study bias) and discuss its likely impact on the results of the review?

For Yes:

- ☐ performed graphical or statistical tests for publication bias and discussed the likelihood and magnitude of impact of publication bias

☐ Yes
☐ No
☒ No meta-analysis conducted

16. Did the review authors report any potential sources of conflict of interest, including any funding they received for conducting the review?

For Yes:	
☒ The authors reported no competing interests OR	☒ Yes
☐ The authors described their funding sources and how they managed potential conflicts of interest	☐ No

To cite this tool: Shea BJ, Reeves BC, Wells G, Thuku M, Hamel C, Moran J, Moher D, Tugwell P, Welch V, Kristjansson E, Henry DA. AMSTAR 2: a critical appraisal tool for systematic reviews that include randomised or non-randomised studies of healthcare interventions, or both. BMJ. 2017 Sep 21;358:j4008.

x
